# Scientific Literacy in Communicating Science and Socio-Scientific Issues: Prospects and Challenges

**DOI:** 10.3389/fpsyg.2021.758000

**Published:** 2021-11-01

**Authors:** Yanyan Li, Mengmeng Guo

**Affiliations:** ^1^School of Management, Zhejiang Shuren University, Hangzhou, China; ^2^College of Literature and Journalism, Sichuan University, Chengdu, China

**Keywords:** scientific literacy, socio-scientific issues, media, bibliometric, public understanding of science

## Abstract

A holistic view of *scientific literacy*-related literature was generated based on bibliometric analysis. The purpose was to provide insight into and knowledge on evolving knowledge fronts and to highlight the turning points in the existing literature between 1980 and 2019. *Scientific literacy* in society can potentially help to answer unsolved riddles of socio-scientific issues (SSI) to enable people to become smart and responsible global citizens. Specifically, two decades before and after the year 2000 was taken into account as it comprised the most noticeable revolutionary developments in terms of economics, technology, culture, and society. Interestingly, the attention paid to *scientific literacy* significantly increased after the financial crisis of 2008. *International Journal of Science Education* and *the Journal of Research in Science Teaching* were observed to be the top-cited and top publishing journals, respectively. Similarly, Jonathan Osborne, Rosalind Driver, and Norman G. Lederman were recorded as the most cited and most published authors, respectively, during the study period. Alarmingly, most of the literature evolved in and was dominated by the Western region, indicating the need to understand the regional-cultural complexities of the East and the rest of the world. The recent evolving clusters, with titles of *literacy (as a concept)*, *learning progression*, and *informal reasoning*, were observed to be currently active knowledge areas in the evolution of the intellectual structure of *scientific literacy*-related literature. However, no recent trend or emerging research direction was noticed in the last decade, even though new and digital media (including immersive media) have revolutionized the communication channels and public understanding of science and socio-scientific issues.

## Introduction

The literal meaning of *literate* is a letter (derived from the Latin word *littera*). Moreover, *scientific* reflects knowledge (derived from the Latin word *scientia*; Rusli, 2012). In 1958, the first traces of *scientific literacy* in the academic literature were observed when the need for public understanding of science was raised (Hurd, 1958). However, *scientific literacy* is currently in the limelight in terms of contemporary education (Laugksch, 2000; Levinson, 2010), civic engagement (Greenhow et al., 2015; Rudolph and Horibe, 2016; Brouwer and Hessels, 2019), and cultural dynamics (Bonney et al., 2009; van Eijck and Roth, 2010). Scientists rarely distinguished *scientific literacy* and *science literacy* in early academic literature (Hurd, 1958; Shen, 1975; Carson, 1997). Conceptually, Victor Showalter (1974) unified the goals of science education through seven dimensions of *scientific literacy*, which comprises the individuals’ ability to understand the nature of scientific knowledge, the capability to accurately apply scientific concepts, efficacy to use processes of science, values with the essence of scientific principles, readiness towards science and technology while viewing society, belief in lifelong learning, and with the readiness to develop science and technology-based skills. Moreover, science and *scientific literacy* are closely related terms in science education research (Roberts, 2007), as *scientific literacy* comprises the positivity appreciating the outcome of science (in terms of education and literacy; Miller, 2004). The reading and writing of science text provoke scientific thinking (scientific inquiry), and its proficiency drives the ability to know science in everyday life, which is the demand of participatory science-based civil society (Podgornik et al., 2017). In the authors’ view, Vision I and Vision II in science education demand an equal level of attention while examining the evolution of academic literature. Specifically Vision I addresses the processes and product of science, and Vision II pays attention to the significant role of situation and environment where a scientific component exists.

The academic literature on *scientific literacy* has become conceptually diverse and substantial. Over the same period, it has expanded and become voluminous (Laugksch, 2000). Critically, the 4th grade slump (Zheng and Warschauer, 2015), lack of acceptance of evolution (Fowler and Zeidler, 2016), weak ability to engage in SSI reasoning (Çalik and Coll, 2012), less readiness for scientific inquiry (Wu et al., 2015), the urge to increase public understanding of science (Bauer et al., 2007), the need for a life-long learning ability (Falk et al., 2007), missing protocols for scientific communication (Bauer et al., 2007), and insignificant interest in society require immediate attention and revisiting of the *scientific literacy* literature. However, in the authors’ view, no comprehensive unified view has been observed which can achieve the goal of *scientific literacy*. The similar standpoint can also be observed in the research contribution by Roberts, 2007, and Roberts and Bybee (2014). The present study intended to predict and understand the development of *scientific literacy*-related literature and to forecast the future trends in the scientific journey to conceptualize a scientific community and responsible citizenship building for the world.

The evolution of the scientific and science literacy literature over the last six decades has left it sufficiently mature as an academic discipline and strategically valuable as a research area to strengthen the workforce, nations, and economies. Thus, the structural evolution of *scientific literacy* in academia can be re-viewed with support of Big Data application for data visualization tools and techniques to underline interesting patterns in the disciplinary growth. The current research was purposefully conducted for the following reasons: (1) to perform a comprehensive review of the intellectual structure of *scientific literacy* with the aid of available data visualization techniques; and (2) to generate a bibliometric view of *scientific literacy* to achieve a better understanding of the knowledge area by highlighting the dominant research contribution, authors, and countries in the literature evolution. In other words, the purpose of the study is to highlight the evolution of *scientific literacy* in terms of research focus (area of the curriculum) as well as the future directions (research fronts) and academic foundations (intellectual bases) of *scientific literacy*.

Previous notable literature analyses of *scientific literacy* include the notable contribution of Laugksch (2000) who surveyed related English literature, and also highlighted different interest groups and their related terms and definitions. Yore et al. (2003) specifically researched the 25-year contribution of the *International Journal of Science Education* in the growth of academic literature of *scientific literacy*. Miller (2004) emphasized scientific literacy in the United States, Roberts (2007) provided a comprehensive view from the perspective of noticeable academic contributors to define the similarities and differences between science and *scientific literacy*, Allum et al. (2008) examined a cross-cultural view, and Roberts and Bybee (2014) argued about the distinctive characteristics of science and *scientific literacy* and the related need of redefining curriculum. However, no initiative has been taken to examine the intellectual structures through visual citation analysis in the concerned knowledge area. Specifically, *intellectual structure* development comprises a four-step procedure. First, nodes (document or author) which received a citation above the predefined threshold are taken under consideration. Second, an algorithm (pathfinder network scaling) is applied which computes the correlation and factor analysis of co-cited nodes. Third, sub-groups (as the outcome of factor analysis) of the knowledge domain through inter-connectivity are computed. Fourth, the citation of the highly cited node within each subgroup is listed to define the nodes’ impact and magnetite in terms of influence within sub-groups (Chen and Paul, 2001). Moreover, the earliest and latest co-citation and its frequency help to gauge potential attractiveness of each node within the intellectual structure (cognitive structure) of the discipline which is labeled as *Burst*. For this purpose, the most user-friendly, standardized, and attested citation analysis software was used, namely, CiteSpace from Drexel University. In particular, this tool is open source and the most renowned in the field of library and information sciences for bibliometric purposes. The distinctive features of the bibliometric approach includes usage of Bradford’s dispersion law to examine literature (Budd, 1988), Zipf’s law to explore growth-pattern (Piantadosi, 2014), and authors and countries’ contribution with the support of Lotka’s law (Pao, 1985) to empirically present *scientific literacy* as the knowledge domain and its literary expansion and growth.

Specifically, bibliometric analysis encourages the understanding of particular phenomena (Hérubel, 1999) in the scientific literature in a quantitative manner (Pritchard, 1969). It encourages the exploration of multiple dimensions of evolving academic research to determine trending methods, models, concepts, and terminologies in the pool of literature (White and McCain, 1989; Narin et al., 1994). It helps to identify knowledge bases and research fronts under the examined intellectual structure of any particular knowledge domain (Li and Zhao, 2015; Mao et al., 2015). Bibliometric analysis of *scientific literacy* enables identification of the dominating nodes (research document, authors, and countries) in the existing literature (Hérubel, 1999; Hood and Wilson, 2001; Wang et al., 2016), and an examination of the evolution of knowledge areas over the timespan of several years (1980 to 2019). Specifically, the noticeable feature of bibliometrics to produce the intellectual structure of discipline makes it distinctive and preferred in contrast to other approaches of literature reviews, i.e., meta-analysis and best-evidence synthesis. The current study aimed to utilize data visualization and network analysis tools to generate an unbiased and comprehensive view of the literature. In further sections, the methodological aspects and detailed results of the study are discussed. This paper also includes a discussion of the findings of the bibliometric results to emphasize future directions and emerging trends.

## Methodology: Bibliometric Analysis

Quantitatively, the bibliometric analysis enables researchers to view scientific knowledge with the ability to determine emerging patterns and their evolution (Pritchard, 1969). The holistic view of disciplinary knowledge evolution through bibliometrics helps to identify knowledge fronts from the intellectual bases of disciplinary knowledge. Interestingly, the rapid pace of multi-disciplinary knowledge evolution has contributed significantly to providing a scientific view of social and environmental problems, although most of the applied research focuses on specialized, narrow research gaps to penetrate intellectual knowledge bases (Swanson, 1993). Thus, the specialization trend allows for vibrant but blurred trends and multi-disciplinary overlaps as gaps in research with the potential to create bursts in knowledge evolution. The bibliographic initiative encourages the examination of disciplinary knowledge to uncover the logical, vital, and untouched structures in the form of dominant participants [i.e., authors, journals, keywords, and research articles (White and McCain, 1989)], which is one of the purposes of the current study. Strategically, this initiative provides an opportunity to magnify micro-level structures and to analyze the corpus of disciplinary knowledge in the form of links and nodes and the nature of associations between them (Narin et al., 1994). Specifically, the co-citation examines the co-occurring trend of two nodes (articles) together in the pool of academic literature. By performing a co-citation analysis of articles in the field of *scientific literacy*, a co-cited article-based cluster view of the intellectual structure was generated to achieve one of the primary purposes of the current study.

The data visualization and network analysis was performed with the support of CiteSpace. It is a Java-based data visualization tool, which supports Big Data analysis (BDA; Yang et al., 2017). With the recent development of the application, it now has more compatibility with the world’s leading indexing bodies (e.g., Thomson Reuters’s Web of Science, Scopus). CiteSpace easily encourages network extraction as it uses the ‘minimum spanning tree, pathfinder, and expectation–maximization’ algorithm with time-slicing features (Yang et al., 2017). Previously, CiteSpace was used to examine intellectual fields and how knowledge fronts evolve (Chen et al., 2016; Asmi et al., 2018; Anwar et al., 2019).

During the preliminary phase of data collection and analysis, all of the contributing authors inspected the crawled data against the search query by reviewing the titles, abstracts, and keywords of each of the crawled records. During the manual examination phase, the crawled data records were examined in the relational grid view to generate possible intellectual structures. The process to generate an understandable intellectual crux was achieved by performing the following operations: (1) the co-citation of nodes (articles and authors) using the data visualization tool (CiteSpace); and (2) the co-occurrence of nodes (countries) observed in the crawled data through the data patterns and trend identifiers (CiteSpace). In summary, the bibliometric analysis initiative reveals the intellectual bases, knowledge fronts, and dominant contributing nodes (i.e., scientists, documents, countries, and geographic regions). The current study followed the methodology previously used in studies of a similar nature (Chen et al., 2016; Madani and Weber, 2016; Anwar et al., 2019).

In the current study, metadata from the academic articles published in Scopus were extracted, as these data comprise the most valued and highest quality research on science and *scientific literacy*. After reviewing the pre-analysis settings, a customized search query was used to explore and extract data from the Scopus database. The searching filter was as follows: ALL (“Scientific Literacy”), where ‘ALL’ was defined as all fields to be searched (including abstract, title, keywords, etc.). To render the search results relevant and reliable, further filters were added to exclude all reviews (588), notes (86), editorials (85), letters (30), short surveys (18), conference reviews (7), and errata (3) as suggested by the previous studies (Gu et al., 2017). Authors’ intention to exclude reviews, letters, editorials, and short surveys as a purpose of the current study is to examine the intellectual growth of the related academic literature. However, reviews and brief documents (i.e., letters) are labeled as noise in bibliometric studies (Li et al., 2017). Moreover, authors also excluded the nodes (research document) which hold no bibliographic details, as suggested by previous studies (Seyedghorban et al., 2016). The final count of 9,578 (Open Access: 1487, Others: 8,100) bibliographic records from the years 1980 to 2019 were collected in the third quarter of the year 2020.

**Figure 1:**
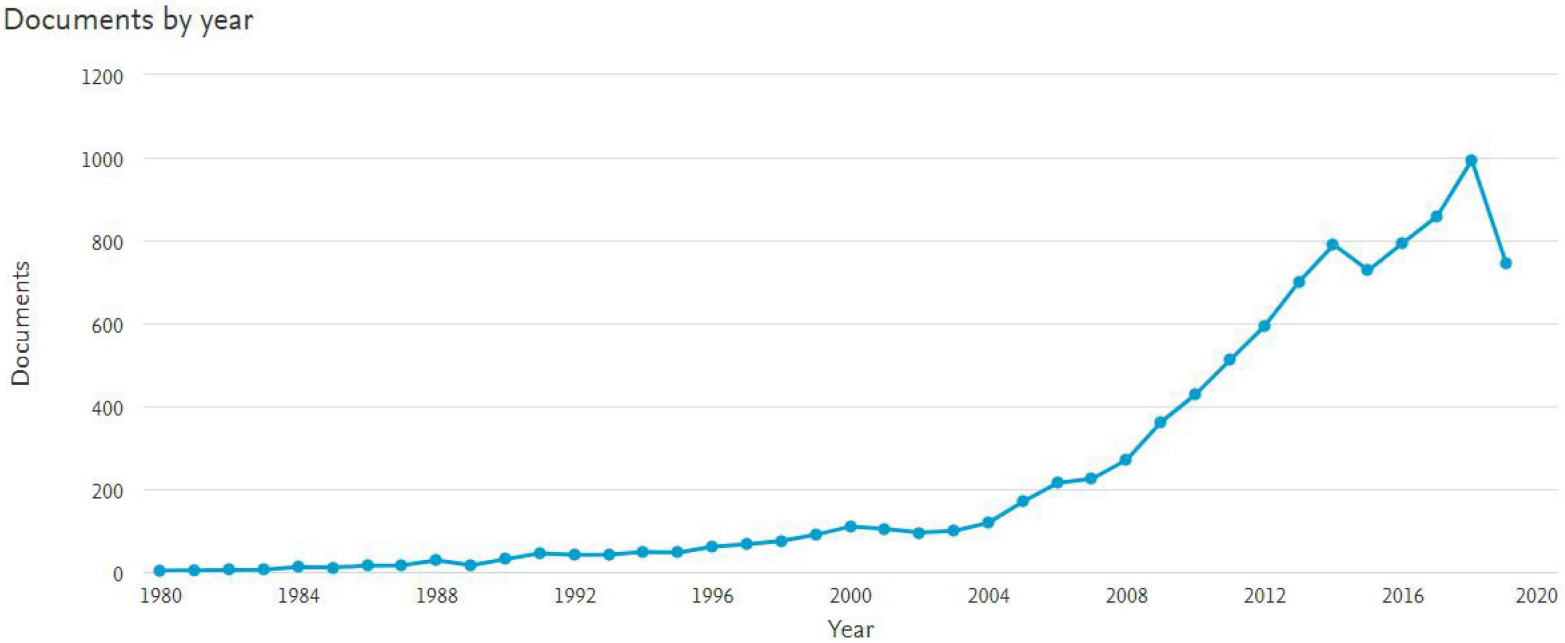
Total publications per year in the field of Scientific Literacybetween the year 1980 to 2019.

CiteSpace was used to help examine the intellectual fields and the knowledge fronts as they evolved (Zhang et al., 2015; Chen et al., 2016), to diversify the literature on *scientific literacy*. The trends in publications and citations were used to evaluate and gauge the importance and popularity of *scientific literacy*. In terms of the publication count for each year, as shown in Figure 1, a sudden growth of *scientific literacy* occurred in the decade since the global financial crisis.

**Table 1:** Top 5 article co-citation bursts in the literature of scientific literacy between the years 1980 to 2019.

Authors and Year	Burst Size	Title	Highlights
Dickinson et al., 2012	38.74	The current state of citizen science as a tool for ecological research and public engagement	Strategic use of citizen science for socio-scientific issues to be addressed, public awareness and education, to appreciate sustainability and to involve non-scientists in scientific research
Roth and Barton, 2004	36.27	Rethinking Scientific Literacy: From Science Education as Propaedeutic to Participation in the Community	Urge to have community participation to build previously uninterested lifelong learning
Bell et al., 2009	28.39	Learning Science in Informal Environments: People, Places, and Pursuits	Importance of learning science from the informal environment, the impact of venue and configuration of the learning environment, and the critical role of media.
Sadler, 2004	27.61	Informal Reasoning Regarding Socio-scientific Issues: A Critical Review of Research	The significance of the relationship between Nature of Science and socio-scientific issues-related decisions, use in curriculum, and argumentation.
Duschl et al., 2007	27.47	Taking Science to School: Learning and Teaching Science in Grades K-8	Examined K-8, and concluded to fundamentally revisit science education to improve foundations with the use of History and Philosophy of Science

## Results

The current section comprises quantitative findings on 1948 authors, representing 135 countries, with 159 funding agencies and 158 academic journals observed to have participated in the evolution of the academic literature related to *scientific literacy*. Over the last 40years, 27 different subject areas were recorded while discussing *scientific literacy*. Specifically, 7,752 records, with a total citation count of 381,159 highlighted the academic worth of the knowledge area of *scientific literacy* to be examined and to be sketched intellectually and structurally. To maximize the understanding of the intellectual structures of *scientific literacy* in the sphere of intellectual growth, institutes, journals, and funding sources were examined. From the primary data analysis, a few interesting trends can be observed. For example, more than 70% of the institutions, which share the top 15 contributors, are from North America. However, very few representatives across the globe succeed to mark their presence, i.e., Universitas Pendidikan Indonesia (Indonesia), the University of Oslo [Norway, Curtin University (Australia), and Nanyang Technological University (Singapore)].

While examining the contribution of journals in the growth of *scientific literacy*-related literature, *International Journal of Science Education* (498), *Journal of Research in Science Teaching* (321), *Science Education* (281), *Research in Science Education* (249), and *Science and Education* (172) were noted as the most active contributors to the literature related to *scientific literacy*. Interestingly, 1,521 research documents by these top 5 contributed journals comprised the subject area of Social Science (84.5%) and Art and Humanities (15.5%).

**Figure 2:**
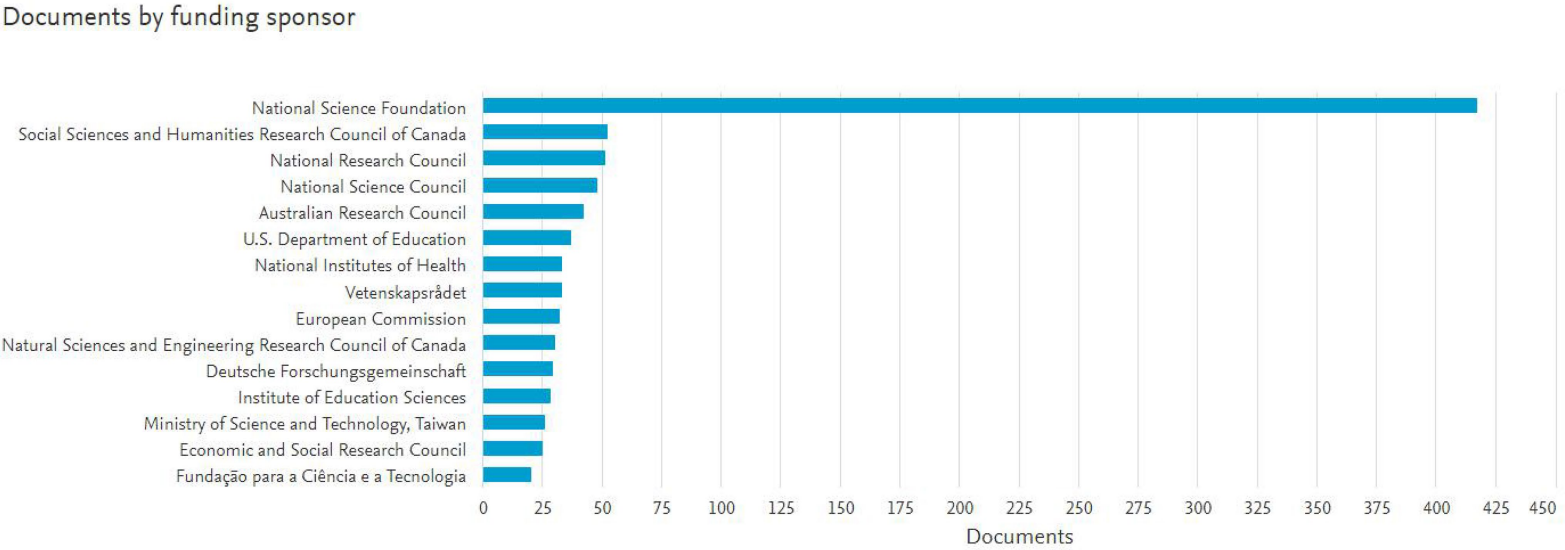
Top 15 funding bodies as contributors in the field of scientific literacy between the years 1980 to 2019.

It is noticed that 50% of the funding institutions belong to North America. The other funding institutions include the Australian Research Council (Australia), Vetenskapsrådet (Sweden), European Commission, Deutsche Forschungsgemeinschaft (Germany), Ministry of Science and Technology (Taiwan), Economic and Social Research Council (United Kingdom), and Fundação para a Ciência e a Tecnologia (Portugal) as shown in the Figure 2.

To perform intellectual structural analysis, a co-citation analysis of academic publications and authors was performed. Moreover, the co-occurrence of country was analyzed. The intellectual turning points, most valued articles, and perdition and understanding of the existing and upcoming research fronts were usually driven by the co-citation analysis of the articles. Moreover, the micro- and macro-level structural evolution was examined through the authors’ analyses, respectively.

In terms of evolution, the divergence in socio-economic environments always triggers and initiates new interdisciplinary dimensions to better understand social and economic events (Cohen and Lloyd, 2014). The purpose of the following sub-sections is to systemically evaluate the dynamics of this evolution.

## Article Co-Citation Analysis

It is important to examine the co-cited references and ensure understanding of their relevancy and the networks among them (Chen and Ibekwe-sanjuan, 2010). Such an examination comprehensively provides the primary structure of the academic research area in an intellectual manner (Chen, 2004). The trends in citations help to identify the associations with the research field (Chen, 2006b). The leading trends in the citation of any specific article indicate the importance of the cited article in the literature (Wang et al., 2014). In the case of *scientific literacy*, 1,648 nodes and 1907 links were identified in the examination of cited references, with the top 50 per slice being initially fixed as the selection criterion. CiteSpace usually considered the rounded shape node as a single cited entity (i.e., author, article, or journal; Chen, 2006a). The size of each circular node used represents the citation frequency over time (Chen et al., 2012). Similarly, the thinness of the links between two nodes usually indicates the citation frequency (Chen et al., 2012). In other words, a high frequency of co-citation is represented by a thick line between nodes (Chen, 2016).

**Figure 3:**
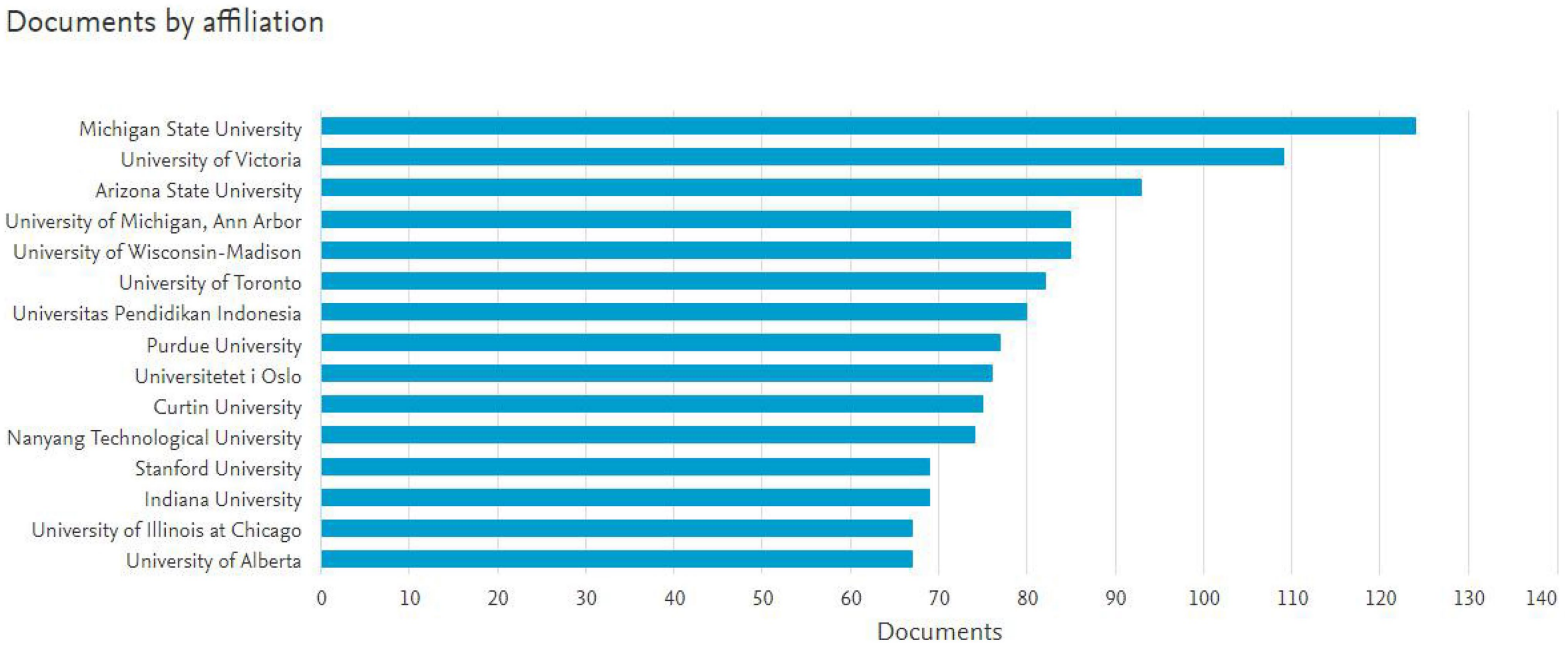
Top 15 institutions as contributors in the field of scientific literacy between the years 1980 to 2019.

In Figure 3 below, the top highly co-cited articles are shown. Specifically, the node by Bonny and his colleagues (2009) entitled as *Citizen Science: A developing tool for expanding science knowledge and Scientific Literacy*, with a co-citation count of 333, where the authors are emphasizing the importance of citizen science to increase scientific knowledge was the highest. It further proposes a model to build and operate citizen science projects. The second highest co-cited document is by the National Research Council’s (1996) *National Science Education Standards* as a vision of scientifically literate populace, with a co-citation count of 162 recorded. It discussed the National Committee on Science Education’s efforts to standardize and assess science teaching (in six levels), professional development of science teachers (in four levels), assessment of science education (in five levels), and standardization of science content. The third highest cited document is written by Osborne and Dillon (2008) with the published title of *Science Education in Europe: Critical Reflections* (co-citation=136), and they questioned science education in Europe (including improving curriculum, pedagogy, assessment, and teaching supply).

Moreover, they proposed seven recommendations to improve science education (about quality of teachers, assessment of science education, improve engagement, engage experimentation, a career in science, innovative curricula, and updating major explanations and the material world). The fourth highly co-cited reference noted was National Research Council's (2013), *‘Next Generation Science Standards (NGSS): For States, By States’* with a co-citation count of 125. The document examined the NGSS’s consistency with the previously defined vision of K-12 science education (framework) and required changes. In the fifth position with a co-citation count of 123, another document by the National Research Council (2011) as NGSS –a 3D view (comprising practice, crosscutting concepts, and disciplinary core ideas) for K-12 science students was presented. The sixth highest co-cited document noted had the title *Scientific Literacy/Science Literacy* as a book chapter by Roberts (2007) holding a co-citation count of 114. It explored in-depth differences, similarities, and assessments with defining and discussing *scientific literacy* by whom, for whom, and their justification of argumentation.

The seventh highest co-cited document written by Silvertown (2009) with the published title of *A new dawn for Citizen Science* had a co-citation count of 109. The author discussed citizen science as a strategic tool to increase public engagement, accountability, and the sense of free labor. In the eighth slot of the highest co-cited articles, a document by Norris and Phillips (1994) with the title *Interpreting pragmatic meaning when reading popular reports of Science* had a co-citation count of 103. This concluded based on 91 12th grade students’ experiments that students are failing to interpret the pragmatic meaning of news reports and failing to accurately understand the scientific status in news in media. In ninth position, Cavagnetto’s (2010) contribution with the title of *Argument to foster Scientific Literacy: A review of argument interventions in K-12 Science contexts*, with a co-citation count of 87, emphasized the significance of *scientific literacy* as it provokes argument-based intervention in science education, and explained the wide spectrum of orientations while discussing the nature of argumentation. The tenth most co-cited reference is by Doulas Allchin (2011), with the published title of *Evaluating knowledge of the nature of (whole) science* and a co-citation count of 84. He analyzed the methods to assess the nature of knowledge, with the intentions to transform individuals from declarative to functional, and interpretative into more critical with the ability to profile key information without it being stated.

## Evolutionary Hotspots in the Literature

The following portion of the co-citation investigation through CiteSpace was performed to identify the evolutionary turning points in the literature over the specified period (Zhang et al., 2015). The node (article) can be represented as evolutionary because it connects numerous nodes. During the reference co-citation analysis through CiteSpace, the highlighted nodes with centrality (between-ness) can be seen as shown in the figure (Chen, 2006b; Chen et al., 2012). In other words, centrality behaves like a bridge connecting several time zones in the developmental pace of the intellectual structure of the knowledge area (Chen et al., 2016). Specifically, it includes the linked chain of (1) Bauer (1992), with a centrality of 0.50, who highlighted the importance of science and technology in modern life. Furthermore, they stated that the misconceptions related to Nature-of-Science and scientific activity are depreciating the use of science in social activities; (2) Shamos (1995), with a centrality of 0.50, who emphasized the less effectiveness of existing educational reforms and urges the increase of science awareness. Specifically, Shamos highlighted (1) the expected value to be produced by science education, and (2) that science is a technique to acquire knowledge, and existing curriculum evaluation methods are less fruitful; (3) Eisenhart et al. (1996), with a centrality of 0.90, discussed how low scientific knowledge, less effective teaching in school, low participation of minorities and women, and less use of science in decision making are obstacles in *scientific literacy* in society; and (4) Driver et al. (2000), with a centrality of 0.90, underlined the lacking of argumentation in scientific controversies among students, as students hold weak oppositional frameworks. Moreover, they concluded the weak pedagogical expertise among teachers as the core reason of low *scientific literacy* among students.

## Article Co-Citation Burst Analysis

CiteSpace provides a ‘burst detection’ algorithm, which simplifies the process of identifying hotspots in the intellectual structure of the literature (Chen et al., 2012). In other words, ‘knowledge fronts’ are used to retrieve the evolving intellectual bases of the knowledge area through ‘burst detection’ (Chen and Ibekwe-sanjuan, 2010; Seyedghorban et al., 2016; Zheng et al., 2016). Burst detection assists in emphasizing the articles that are cited intensively during a specific time frame (Chen, 2004, 2006b; Lin et al., 2015). Specifically, Kleinberg’s (2003) algorithm was adopted, and it inspects the transient nature of research fronts to identify bursts (Chen, 2006b) in the field of *scientific literacy*. Apart from the contribution made by the highly cited contributors as discussed above, the documents by Dickinson et al. (2012), Roth and Barton (2004), Bell et al. (2009), Sadler (2004), and Duschl et al. (2007) with a high score burst value are shown in the Table 1.

## Cluster Analysis

CiteSpace analyses helped to closely group associated cited references and identify weak bonds with less relevant members (Chen, 2006b). The article co-citation network enabled identification of the cluster labels by analyzing the titles, abstracts, and keywords of each of the selected documents. The citation trends through CiteSpace usually follow mathematical algorithms, e.g., Latent Semantic Indexing (LSI), which usually follows a dimension reduction strategy (Wei et al., 2015; Li et al., 2016); Log-Likelihood Ratio (LLR), which is commonly used to measure the goodness of fit by comparing two models derived from the likelihood ratio (Li et al., 2016); and Mutual Information (MI), which, in the context of information theory, explains one term on the basis of the random occurrence of another term to understand the dependencies (Chen and Ibekwe-sanjuan, 2010; Li et al., 2016). However, the labels derived from LLR are preferred because they are used to provide a more comprehensive view of the intellectual network structure (Wei et al., 2015; Li et al., 2016). The term ‘silhouette’, as the output of the cluster analysis of citation networks, is used to explain the homogeneity within the cluster. Specifically, a higher silhouette value explains a higher degree of consistency among the references shared in the cluster (Kozlov et al., 2015; Yu and Xu, 2016).

**Figure 4:**
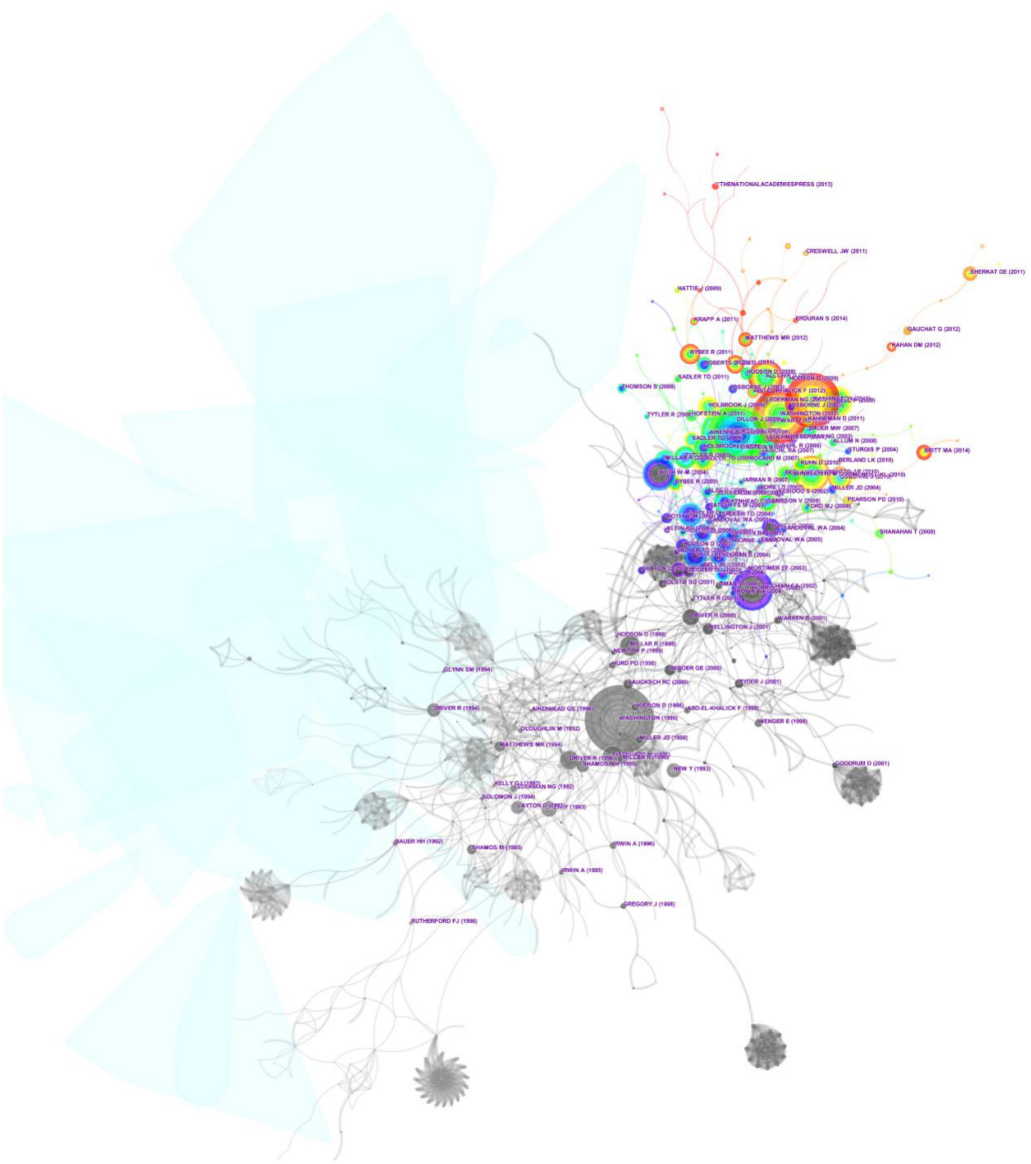
Highly co-cited articles in the intellectual field of scientific literacy between the years 1980 and 2019.

The re-visited intellectual base in the cluster view encourages identification of co-citations using different cluster-labeling algorithms, i.e., LLR, MI, and LSI. Moreover, it helps to classify co-citations and cross-cluster co-citations in a meaningful manner (Chen, 2006a; Chen and Ibekwe-sanjuan, 2010). In the following section, the most influential cited articles in the dominating clusters of the intellectual structure of *scientific literacy* are discussed to obtain a high-level view of the cluster analysis in an explorative manner (Figure 4). Narratively, the top three clusters from the pre and post era of the year 2000 are taken under consideration, as shown in Table 2.

**Table 2:** Six notable clusters in the intellectual structure of scientific literacy pre and post the year 2000 (1980 to 2019).

Mean Year	ID	Size	Silhouette	LSI	LLR	MI
1993	4	53	0.822	Science; science knowledge; cognitive strategy use	Cognition and cognitive apprenticeship; talking their way into science	Cognition and cognitive apprenticeship; marginalized discourses and scientific
1995	3	50	0.810	Science; media; pupils; biomedical communications; reasoning; parents views	Scientific reasoning; misunderstanding science; science and technology	Science and technology; civic scientific literacy
1996	6	33	0.914	Science; nature; instructional practice; competent scientific practice	Social knowledge; cultural basis; information source	Social knowledge; cultural basis; moving toward a portfolio culture
2007	1	96	0.794	Science; nature; evaluating knowledge; curriculum reform; high school	Literacy component; new direction; teaching nature	Equipping student; science model; explicit and reflective versus
2002	2	66	0.885	Science; values; young people; literacy; fundamental sense;	Informal reasoning; society and environment; research on conceptual change	Society and environment; research on conceptual change
2009	7	45	0.871	Science; language; English language learners; argument; online argumentation	Learning progression; scientific argumentation	Argumentation from science studies; epistemic thinking

The order of the clusters is presented according to the cluster size.

**Table 3:** Highly co-cited authors in the intellectual structure of scientific literacy between the years 1980 to 2019 with a threshold of 600.

Frequency	Author	Research focus	Affiliation
1,058	Jonathan Osborne	Curriculum, classroom, argumentation, and women’s participation in science	Stanford University, United States
958	Rosalind Driver	Conceptual Change, Argumentation	Kings College London, United Kingdom
725	Norman G. Lederman	Nature of Science, Scientific Inquiry	Illinois Institute of Technology, United States
683	Rodger Bybee	Secondary School Science, Curriculum	Carleton College in Northfield, Minnesota, United States
668	Derek Hodson	Curriculum and Pedagogy	Ontario Institute for Studies in Education, Canada
628	Miller Jon D	Citizen science, policy, public attitude, biomedical communication	University of Michigan, United States
614	Robin Millar	Socio-scientific issues, Moral reasoning	University of York, United Kingdom
605	Rick Bonney	Scientific Curriculum, Physicists	New York University, United States

**Figure 5:**
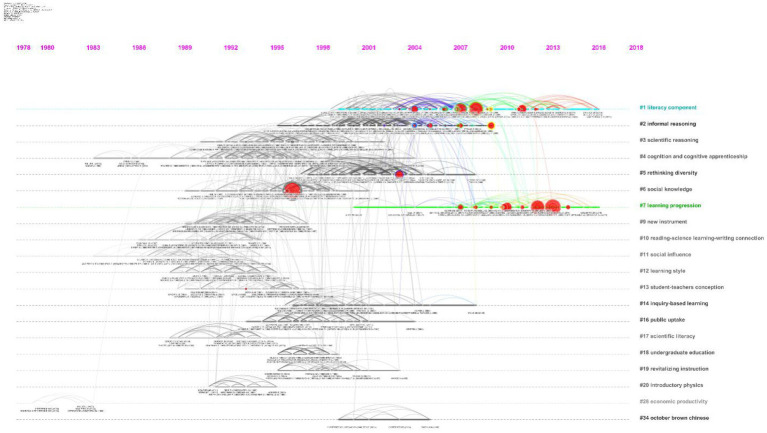
Co-citation-based cluster analysis of the intellectual field of scientific literacy between 1980 and 2019.

Throughout the evolutionary timeline, a triggering cluster with a silhouette value of 0.997, indicating data from 1981 with the LLR label of (#28) ‘economic productivity’ was observed, as shown in the lower-left corner of Figure 5. However, in the present era of the intellectual evolution of *scientific literacy*, clusters with the LLR label of Learning Progression and Literacy Component dominate which will be discussed in detail in the following subsection.

**Table 4:** Authors with highest burst count in the intellectual structure of scientific literacy between the years 1980 to 2019.

Author	Burst	Start year	End year	Timespan	Research Area
Rick Bonney	147.4205	2016	2019	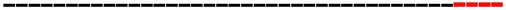	Citizen Sconce
Janis Dickinson	68.4365	2015	2019	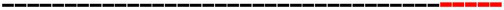	Citizen Science
Jonathan Silvertown	64.1567	2016	2019	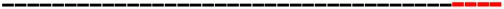	Citizen Science
David Layton	53.55	1990	2003	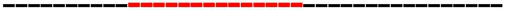	Scientific literacy and Technology
Dominique Brossard	50.6621	2015	2019	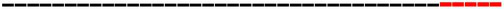	Science Communication, Media

From the era of pre-2000, the clusters with the LLR label of (#4) *Cognition and Cognitive apprenticeship* with a silhouette value of 0.822 from the year 1993 were recorded. Specifically, the prominent contributors were noted such as Rosalind Driver et al. (1994) who stressed the importance of social settings, and culture as a tool to socialize learners while developing scientific knowledge. The other prominent names include Joan Solomon and Glen Aikenhead as they emphasized the role of Science, Technology, and Society (STS), student’s preconception, and cross-cultural barriers in science education. The second prominent cluster was observed with the LLR label of (#3) *Scientific Reasoning* with a silhouette value of 0.810 from the year 1995. This cluster has the greatest number of cited documents by Millar and Osborne (1998) as it comprehensively discussed the failures and successes from the past, expectations of young students from science education, possible content and structures of science curriculum, and its related challenges and problems. Moreover, David Layton and George E. Deboer were also noted as significant contributors while discussing the role of *scientific literacy* to enhance public understanding of science and related implications for science education.

Regarding the evolutionary timeline before the year 2000, the third biggest cluster with the mean year of 1996 and LLR (#6) Social Knowledge holding a silhouette value of 0.914 was recorded. During the cluster examination, besides the contribution by the National Research Council (United States) and Margaret Eisenhart, the research document contributed by Fouad Abd-El-Khalick et al. (1998) was one of the most cited articles in this cluster, which mentioned that conceptualization of NOS for classroom practice should be embedded as a cultural element of teacher preparation. Moreover, the social aspect of scientific investigation demands more attention of teachers.

Since the beginning of the 21st century, the academic literature has evolved, addressing the hurdles and barriers to strategically maximize *scientific literacy*. Specifically, noticeable clusters in the post-2000 era include (#1) *Literacy Component*, (#2) *Informal Reasoning*, and (#7) *Learning Progression (argumentation)*. *Scientific literacy* (as a component)-related literature has had prominent contributions by Osborne and Dillon (2008), Roberts (2007), Allchin (2011), and Roth and Barton (2004). It includes Lederman’s (2007) work underlining NOS as an integral part of *scientific literacy*, where he observed that K-12 students and teachers hold a weak conception of NOS, and conclude explicit and reflective instruction as the preferred way to learn conception of NOS. Moreover, the cluster also comprised the dominating work of Glen Aikenhead (2006) where he emphasized humanistic approaches to science. The *Informal Reasoning* cluster is dominated by the contributions of (1) Zeidler et al. (2005) who discussed cultural, discourse, case-based, and NOS-related issues (as pedagogical importance) to define personal cognitive and moral development through SSI education and to promote functional *scientific literacy*. (2) Bell et al.’s (2009) report encouraged an informal learning environment which can be interactive, support participants to interrupt, involve community-educators, and promote the development of educational tools and material. Furthermore, the cluster includes the work of Tytler (2007), Osborne et al. (2004), Sadler (2004), and Driver et al. (2000). The third cluster from the post-2000 era labeled as *Learning progression (argumentation)* comprises the prominent contribution of the National Research Council’s (2011) and National Research Council’s (2013) documents addressing the K-12 science education framework and states review, Duschl et al. (2007) who examined science teaching at K-8, and Cavagnetto (2010) who argued for improving communication and critical cognitive skills. Moreover, it also includes the work by Sampson and Clark (2008) as he reviewed argumentation (in terms of structure, justification, and content), and Britt et al. (2014) who highlighted the significance of *scientific literacy* to understand scientific information. Britt also mentioned the intrinsic complexity of scientific phenomena, interlinkage of different pieces of information, and rhetorical layout of the text as hurdles to learning from science-related text.

## Authors’ Co-Citation Analysis

In terms of publishing academic literature on *scientific literacy* since the year 1980, the most prominent researchers were Wolff-Michael Roth from the University of Victoria, Canada (68), Ingo Eilks from the University of Bremen, Germany (41), and Brian Hand from the University of Iowa, United States (39). However, during the micro-level analysis of the intellectual structure of *scientific literacy*, the most notable authors with the highest numbers of co-citations were Jonathan Osborne from Stanford University, United States (1058), Rosalind Driver from King’s College London, United Kingdom (958), and Norman G. Lederman from the Illinois Institute of Technology, United States (725).

Through the analysis of 931 nodes and 1,611 links during the co-citation analysis using CiteSpace, Rick Bonney had a burst count of 147.42; Janis Dickinson had a burst count of 68.44; Jonathan Silvertown had a burst value of 64.16; David Layton had a burst score of 53.55, and Dominique Brossard had a burst score of 50.66. Interestingly, all the highest bursts scored by individuals emphasized citizen science, technology, and communication as shown in the table below.

## Country-Level Co-Occurrence Analysis

**Figure 6:**
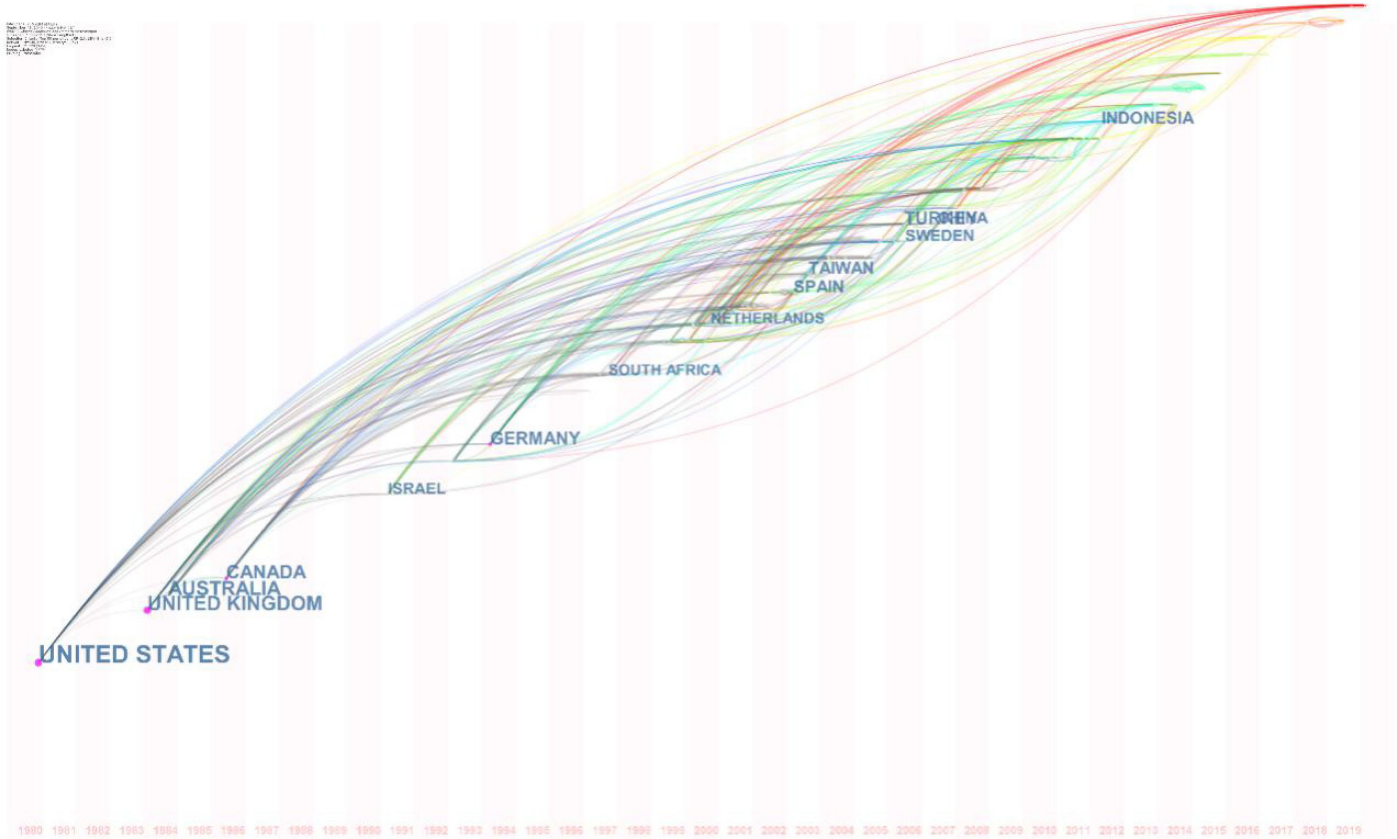
Time zone evolution of intellectual growth of scientific literacy between the years 1980 to 2019.

In the bibliometric approach to analyzing *scientific literacy-*related literature, countries and institutional participation can help to construct macro-level structures of the research field. By the country-level co-occurrence analysis of 135 nodes and 516 links, the United States was ranked first with a frequency of 4,305 and a burst value of 91.76. In other words, the United States accounted for 37.19% of the world’s co-citations during the years 1980 to 2019, followed by the United Kingdom (frequency=749, centrality=0.24), Australia (frequency=724, centrality=0.06), and Canada (frequency=597, centrality=0.18). Interestingly, all the dominating countries in terms of co-citation frequency started contributing to the intellectual structure of *scientific literacy* at the beginning of the 1980s as shown in the Figure 6 below.

The findings conclude that in the recent decade, some of the countries which have never dominated in the literature are finally succeeding in marking their presence (in terms of burst) as shown in the table. Moreover, there are many countries from South America, Europe, and Eastern sphere of the globe which are actively contributing to the literature in recent years as shown in Tables 5 and 6.

**Table 5:** Top 5 country-level bursts in the intellectual structure of *scientific literacy* between the years 1980 to 2019.

Country	Frequency	Burst	Starting Year	Ending Year	Timespan
Indonesia	345	114.0624	2017	2019	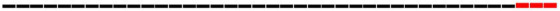
United States	4,305	91.7642	1980	1998	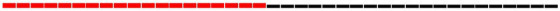
Canada	597	21.3323	1993	2007	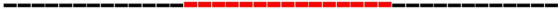
Taiwan	250	18.4882	2011	2015	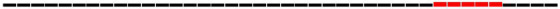
Turkey	382	11.9285	2009	2012	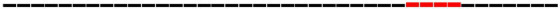

**Table 6:** Ongoing country-level bursts in the intellectual structure of *scientific literacy* between the years 1980 to 2019.

Country	Frequency	Burst	Starting Year	Ending Year	Timespan
Chile	38	5.6422	2015	2019	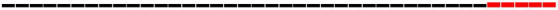
Austria	52	6.1447	2016	2019	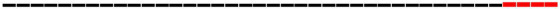
Japan	73	7.4828	2016	2019	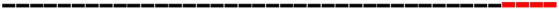
Spain	262	4.2639	2016	2019	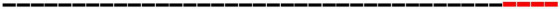
Poland	14	4.2647	2016	2019	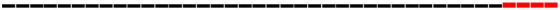
Brazil	117	4.372	2016	2019	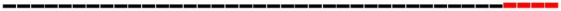
Philippines	8	3.8086	2017	2019	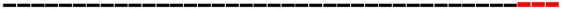

## Discussion

The research work was conducted with the intentions to frame and highlight contributors (articles, country, journal, author, and institutions) through a data visualization technique in the intellectual structure (cognitive structure) of *scientific literacy* in terms of research focus (area of the curriculum) during the last 40years. The main contribution of the current document is to reveal the existing research areas (research fronts) in the discipline through the objective methodology. It further underlines new directions in academic research (by spotting the bursts) in a structured manner. After a quick overview of the intellectual structure, it can be concluded that apart from highly cited articles, documents emphasizing the importance of science and technology in society, challenging the effectiveness of education reforms, and discussing the lack of argumentation can be seen as turning points (centrality). Moreover, informal learning environments, NOS, socio-scientific issues, and citizen science (as bursts) are the most trending attributes in the literature. In terms of the cluster developmental pattern, the findings conclude that *scientific literacy* triggered *economic productivity* as a prime concern, which further provoked the research related to *cognition and cognitive apprenticeship*. It initiated a parallel stream of research related to *informal learning*, *social knowledge*, *instrument development*, and *learning styles* (as dominating clusters). In the present moment, *literacy and its components (as concept and NOS)*, and *argumentation* are noticed as the most valuable sections of *scientific literacy’s* intellectual growth.

A few further interesting findings are as follows: As literature holds a lack of consensus about the definition of *scientific literacy*, the current study highlights that the knowledge background of each of the dominating contributors (authors) hold different educational backgrounds. For example, Joe D. Miller and Rick Bonney are only two researchers in the list of top-cited authors whose research emphasis is public engagement, science communication as policy, and government studies. All other contributors are holding a first degree in physics, biology, chemistry, or other related disciplines. Moreover, authors who have the biggest bursts are emphasizing citizen science, science communication and the role of technology in science education, and *scientific literacy*. In the context of subject areas of literature evolution, the technological aspect is least observed. In other words, literary related to the technology-based environment and its role in *scientific literacy* is less populated. However, it has been researched that motivation to learning about science exerts a mediating effect on technology use, sponsorship of messages (scientist), and trust in the medium (Takahashi and Tandoc, 2016). Hence, the document predicts future subject areas including science communication, and the strategic role of technology could be a potential contributor to the literature of *scientific literacy*.

While exploring the country contributions, Indonesia was observed to be distinctive in terms of burst. However, it is interesting to mention that out of Indonesia’s 345 documents, 213 are conference papers and 266 are Open Access documents, and 70% of the publications were only observed in the last 2years. In contrast, among the top 20 contributing countries, the EU (United Kingdom, Turkey, Germany, Spain, Sweden, Portugal, and Greece) holds 2,209 records (only 123 conference papers, 403 Open Access documents). The purpose of comparing the EU and Indonesia is to predict a sudden growth in the literature, which can bring more challenges in terms of evolving new research fronts in *scientific literacy*.

As shown in Figure 5, through the cluster analysis of co-cited references, it can be concluded that the literature is constantly evolving in relation to *informal reasoning*, *cognitive abilities*, *social knowledge*, *argumentation*, *instruments*, *learning styles*, and *inquiry-based learning*. However, none of the new research clusters evolved in the most recent decade, and technology contributed in each of the clusters. Still, *new technology* in terms of media and communication is struggling to mark a distinctive cluster. For instance, only 138 documents were observed while discussing the role of Augmented Reality (AR) in the academic literature of *scientific literacy*. However, only 24% of them originated from North America. The purpose of arguing is to highlight that new trends and new subject areas are evolving globally. However, the origin of publication is still dominated by North America and it is significantly influencing the appreciation and future evolution of *scientific literacy*-related academic literature. Furthermore, it is important to highlight that 96% of *scientific literacy*-related literature is being produced in English (language). Authors underline that instrumental development, science communication, and *scientific literacy* in an international perspective holds serious challenges for effective and fruitful reforms and development as literature evolution is dominated by a limited pool of origin, language, and funding bodies.

In sum, the evolution of *scientific literacy*’s literature, regardless of research focus, which can be complementary or contradictory to the existing literature, is highly influenced by sponsors’ and authors’ contextual factors (i.e., education, country, and goals).

## Conclusion and Implications

Although researchers have raised crucial questions for future research, the present study highlights the following trends which can be predicted in the future.

The study highlights that during the last 20years, *scientific literacy*’s instrument development in the context of formal and informal education has rarely been distinctively examined by academicians, educators, and policymakers (the evolution of literature can be seen in Figure 5).

The role of *scientific literacy* is to communicate SSIs that exist in literature. However, the strategic use of *scientific literacy* while proposing mitigation or coping strategies for SSIs is still lacking. In other words, *scientific literacy’s* relatedness to behavior modeling (persuasive psychological modeling) is needed.

Among the most populous regions of the globe, responsible citizens’ behavior and resilient community development are critically important. In other words, diversity of culture demands that the attributes of *scientific literacy* be revisited in emerging economies, as it can be clearly stated that *Social Influence* was one of the initial research fronts of *scientific literacy* in the western region of the globe (the same trend can be predicted in the eastern sphere of the globe).

The technological revolution has transformed the medium of communication (i.e., immersive media), its critical role in learning styles, and individuals’ cognitive abilities, while delivering and communicating NOS and concept of science (as a part of literacy components) hold potential to be game-changers in the future. At the same time, technological advancements also have a dark side, i.e., Google-effect, which is affecting humans’ cognitive abilities and argumentation abilities.

Moreover, the current initiative holds implications for academicians and policymakers. Noticeably, academicians can use the current research to highlight and understand the evolution of academic literature. The curriculum under the contemporary settings of *scientific literacy* do enhance the public understanding of science. However, there is a dearth of wanting to transform *scientific literacy* into scientific self-efficacy, which can bridge together the construct-level differences of Visions I and II. This transformation both in academic and policy frontiers could be smooth through the strategic use of immersive media. Furthermore, the concerned policy makers can also propose, design, and re-visit the issues related to science curriculum, public understanding, and science communication in light of an explored intellectual structure. This study concludes that in the recent slice of time, Vision II is getting more attention by academicians and dominating the literature evolution (as compared to the rest of the aspects) within the intellectual structure of *scientific literacy*, as observed nodes related to Vision I in intellectual structure are getting diffused with more inclusion when compared to Vision II, which directs the need and emphasis of academicians towards a scientifically literate populace. The study observed convergence of *citizen science* and *technology-*based research fronts within the literature of *scientific literacy* focusing on SSI (particularly comprised of ecological and environmental concerns). This converging trend of citizen science and technology should include other phenomena related to sustainable behaviors (i.e., GMO, energy consumption, and green modes of mobility), which can help citizens to become smarter and responsible stakeholders of sustainable society.

In terms of Victor Showalter’s (1974) view about society as a dimension of *scientific literacy*, authors argue that domination of North American nodes (i.e., authors, institutions, and countries) are depicting skewness in the intellectual structure of *scientific literacy*, which is also underlining the domination of specific cultures and regions. In other words, existing literature related to instruments of development, argumentation, components of literacy, social knowledge, and influence from the rest of the world is less observable (i.e., BRICS which comprise almost 40% of the world’s population is rarely observed in contributions in the intellectual structure of *scientific literacy)*. Moreover, in a global view, the contribution by the eastern sphere (i.e., China, Taiwan and Japan) is getting distinction in terms of scientific research studies and nation’s IQ, regardless their less significant contribution in *scientific literacy’s* intellectual structure. The above argument emphasizes the need to conduct cross-cultural research which can help to (re) align the construct level conceptualization of *scientific literacy*, and also encourage contributors from the rest of the world to participate in the literature.

Strategic stakeholder management in the case of developing *scientific literacy* is rarely observed in literature. In the authors’ view, a comprehensive framework for the active mode of communication and synchronization among educators, policy makers, and concerned public offices is needed to efficiently design, test, and deliver research-proven and standards-based science curricula. Indeed, for the prosperity and sustainable growth of our planet, *scientific literacy* is a threshold regarding competencies for every human. However, the ‘soft challenges’ (i.e., ‘cultural openness’, ‘digital divide’, ‘religious beliefs’, and ‘skilled and knowledge-full human resource’), tangible resources (teaching instruments, curricula, and resources), and institutional forces (governing bodies to design and implement policies and structures for educational infrastructure) have encountered a new spectrum of ‘literacy’ (i.e., information literacy, media literacy, research literacy, and critical literacy), opening up a large range of interdisciplinary challenges and research directions for all stakeholders in *scientific literacy* (i.e., educators, teachers, and researchers).

## Data Availability Statement

The original contributions presented in the study are included in the article/supplementary material, further inquiries can be directed to the corresponding author.

## Author Contributions

YL: conceptualization, funding, acquisition, supervision, and writing. MG: formal analysis, investigation, methodology, software, validation, and visualization. YL and MG: writing - review and editing. All authors contributed to the article and approved the submitted version.

## Conflict of Interest

The authors declare that the research was conducted in the absence of any commercial or financial relationships that could be construed as a potential conflict of interest.

## Publisher’s Note

All claims expressed in this article are solely those of the authors and do not necessarily represent those of their affiliated organizations, or those of the publisher, the editors and the reviewers. Any product that may be evaluated in this article, or claim that may be made by its manufacturer, is not guaranteed or endorsed by the publisher.
